# Out-of-Frame T Cell Receptor Beta Transcripts Are Eliminated by Multiple Pathways *In Vivo*

**DOI:** 10.1371/journal.pone.0021627

**Published:** 2011-07-13

**Authors:** Grace K. Mahowald, Michael A. Mahowald, Clara Moon, Bernard Khor, Barry P. Sleckman

**Affiliations:** Department of Pathology and Immunology, Washington University School of Medicine, St. Louis, Missouri, United States of America; National Institute on Aging, United States of America

## Abstract

Non-productive antigen receptor genes with frame shifts generated during the assembly of these genes are found in many mature lymphocytes. Transcripts from these genes have premature termination codons (PTCs) and could encode truncated proteins if they are not either inactivated or destroyed by nonsense-mediated decay (NMD). In mammalian cells, NMD can be activated by pathways that rely on the presence of an intron downstream of the PTC; however, NMD can also be activated by pathways that do not rely on these downstream introns, and pathways independent of NMD can inactivate PTC-containing transcripts. Here, through the generation and analysis of mice with gene-targeted modifications of the endogenous T cell receptor beta (*Tcrb*) locus, we demonstrate that in T cells *in vivo*, optimal clearance of PTC-containing *Tcrb* transcripts depends on the presence of an intron downstream of the PTC.

## Introduction

Lymphocyte antigen receptor chains are composed of N-terminal variable regions encoded by the first two exons of the antigen receptor gene and C-terminal constant regions encoded by the remaining exons [1]. The second exon of all lymphocyte antigen receptor genes is assembled by the V(D)J recombination reaction, which is initiated when the RAG endonuclease introduces DNA double strand breaks at the border of recombining variable (V), diversity (D) and joining (J) gene segments and their flanking RAG recognition sequences (recombination signals, RSs) [2]. These DNA double strand breaks are repaired by the non-homologous end-joining (NHEJ) pathway, joining the V, D and J gene segments [1]. NHEJ is imprecise and the random gain and loss of nucleotides that accompanies this joining process is essential for antigen receptor gene diversification and adaptive immunity. However, this diversification also leads to reading frame shifts and premature termination codons (PTCs) in two out of the three reading frames. These out-of-frame antigen receptor alleles are actively transcribed in lymphocytes, providing a rich source of PTC-containing transcripts that if not inactivated or destroyed by nonsense-mediated decay (NMD) could lead to the production of truncated antigen receptor peptides that could be deleterious to developing lymphocytes. Consistent with this notion the selective ablation of Upf2, a central mediator of NMD, in thymocytes leads to defects in T cell development, which could be due, in part, to the accumulation of T cell receptor gene transcripts with premature termination codons [3].

In lower eukaryotes, NMD is activated when PTCs are sensed based on their distance from the poly(A) tract [4]. In contrast, mammalian cells can activate NMD through pathways that recognize termination codons as premature if they lie upstream of an excised intron, irrespective of their distance from the poly(A) tract [5], [6], [7], [8]. Activation of this pathway, termed EJC-dependent NMD, requires that exon junction complexes (EJCs) exist downstream of ribosomes stalled at PTCs during the first round of translation [8], [9]. EJCs are deposited ∼20–24 nt upstream of an exon-exon border, and in order for a PTC to activate NMD by EJC-dependent pathways, it must lie at a distance of greater than 50–55 bp upstream of the exon-exon junction [7], [9], [10], [11], [12].

Transcripts that have PTCs lying less than 50 bp upstream of an exon-exon junction or that have PTCs in the final exon are still able to activate NMD in mammalian cells [5], [11], [12], [13], [14]. Activation of NMD by this fail-safe pathway is thought to require sensing the distance of the PTC from the poly-A tract, as is the case in lower eukaryotes [15]. Although activation of fail-safe NMD does not rely on the presence of an intron downstream of the PTC, it requires the presence of an intron elsewhere in the gene [11], [14], [16], [17], [18]. In addition, mechanisms that, for example, alter splicing can inactivate PTC-containing transcripts without leading to their degradation [19], [20], [21], [22], [23], [24], [25], [26]. In this regard, pre-mRNAs with PTCs can accumulate un-spliced, or incompletely spliced, in the nucleus. Moreover, PTC-containing transcripts can be alternatively spliced removing the exon with the PTC, a process termed nonsense-associated altered splicing. How PTCs are sensed in these incompletely processed transcripts is not completely understood. Together, these mechanisms prevent transcripts with PTCs from encoding truncated proteins that could have detrimental effects.

T cell receptor beta (*Tcrb*) chain locus transcripts containing PTCs are readily destroyed by NMD *in vivo* [27]. Here, we develop an approach to directly determine the requirement for an intron downstream of the PTC in efficiently clearing transcripts templated by the endogenous *Tcrb* locus in thymocytes. Using multi-step gene targeting, we generated two minimally modified versions of the mouse *Tcrb* locus, *Tcrb^A^* and *Tcrb^F^*, that undergo normal rearrangement and expression in developing thymocytes. These two alleles are identical except that *Tcrb^A^* allele PTCs will have downstream introns whereas *Tcrb^F^* allele PTCs will not. By comparing the stability of PTC-containing *Tcrb^A^* and *Tcrb^F^* transcripts in thymocytes, we demonstrate that the normal clearance of these transcripts relies on the presence of introns downstream of the PTC.

## Results and Discussion

### Generation of the *Tcrb^A^* and *Tcrb^F^* alleles

**Figure 1:**
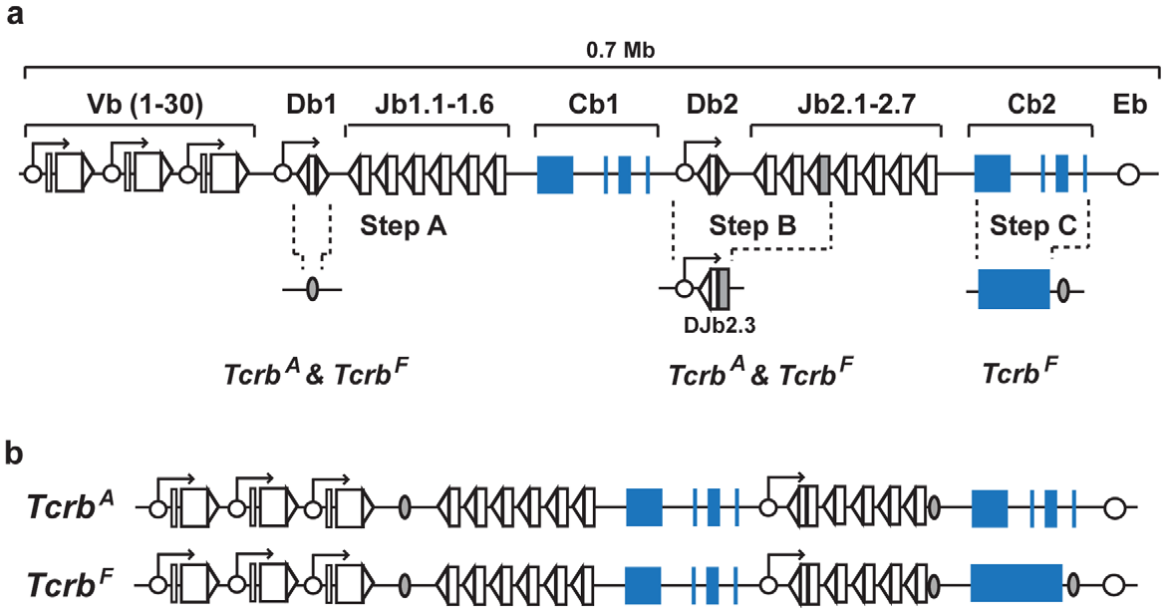
*Tcrb^A^* and *Tcrb^F^* alleles. (**a**) Schematic of the mouse *Tcrb* locus. Vb, Db and Jb gene segments (open boxes), RSs (open triangles), Cb1 and Cb2 exons (blue boxes), enhancer (Eb and promoters (open circles) and *loxP* sequences (shaded ovals) are shown. (**b**) Schematics of the *Tcrb^A^* and *Tcrb^F^* alleles.

The mouse *Tcrb* locus spans 0.7 Mb, with 34 Vb gene segments lying in a 0.4 Mb region upstream of two Db-Jb clusters, each with a single Db and 6 or 7 Jb gene segments (Fig. 1a) [28]. The second exon is completed when a Vb gene segment rearranges to a DJb rearrangement at either of the Db-Jb clusters. Four constant region exons lie downstream of each Db-Jb cluster (Cb1 and Cb2) (Fig. 1a). VDJb rearrangements to DJb1 are transcribed with the four Cb1 exons; likewise, VDJb rearrangements to DJb2 are transcribed with the four Cb2 exons.

Multi-step gene targeting was used to generate mice with two modified endogenous *Tcrb* loci (*Tcrb^A^* and *Tcrb^F^*, Fig. 1b). Initially, the Db1 gene segment was deleted, limiting rearrangement of Vb gene segments to the Db2-Jb2 gene segment cluster (Step A, Fig. 1a, see Jb1^M3^ allele in ref. [28]). The Db2, Jb2.1, Jb2.2 and Jb2.3 gene segments were then replaced with a DJb2.3 rearrangement in its native configuration to generate the *Tcrb^A^* allele (Step B, Figs. 1a and S1). The *Tcrb^F^* allele is identical to the *Tcrb^A^* allele except that the four Cb2 exons have been replaced with a DNA fragment containing a fusion of these exons without any introns (Step C, Figs. 1a and S2). Importantly, as the *Tcrb^A^* and *Tcrb^F^* alleles were generated through modest gene targeted modifications of the wild type endogenous *Tcrb* locus, the *Tcrb^A^* and *Tcrb^F^* alleles remain under the same *cis*-acting regulation (endogenous *Tcrb* promoters and enhancers) as the wild type *Tcrb* locus.

### T cell development in *Tcrb^A/A^* and *Tcrb^F/F^* mice

**Figure 2:**
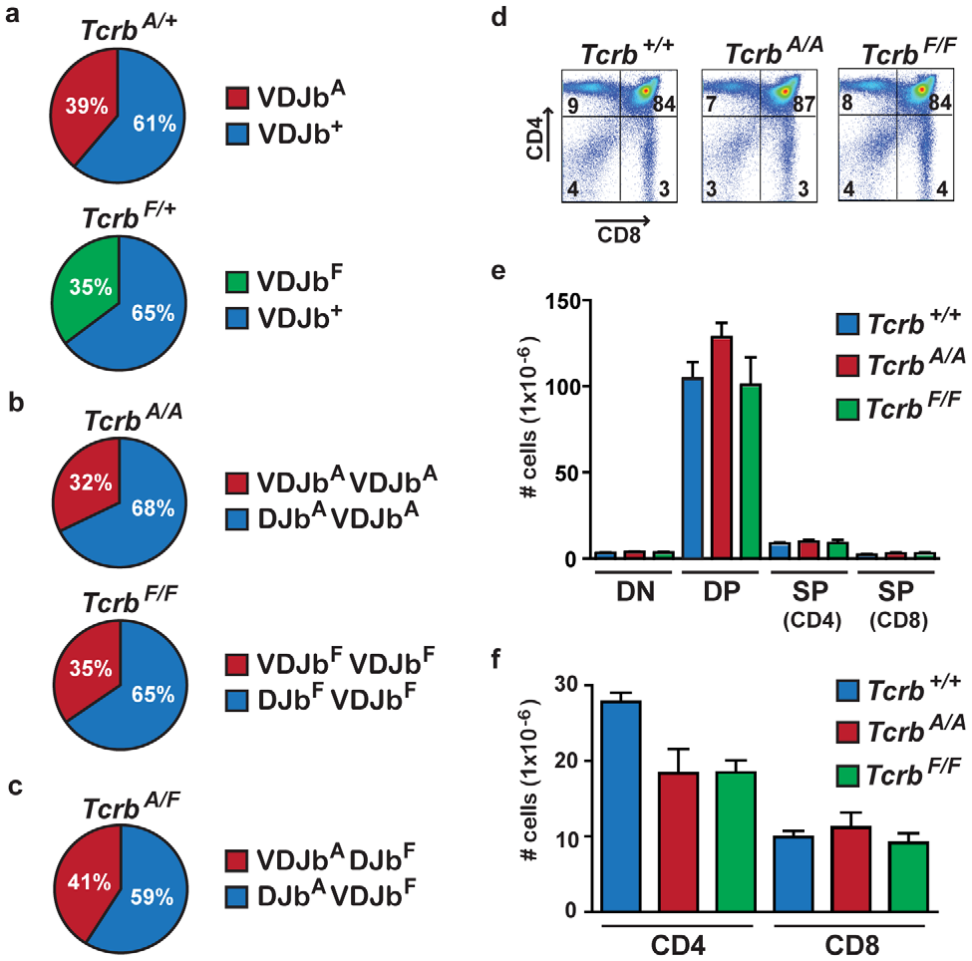
T cell development in *Tcrb^A/A^* and *Tcrb^F/F^* mice. (**a**) Percent of *Tcrb^A^* (red), *Tcrb^F^* (green) and *Tcrb^+^* (blue) alleles in the VDJb configuration in *Tcrb^A/+^* and *Tcrb^F/+^* hybridomas. (**b**) Percent of *Tcrb^A/A^* and *Tcrb^F/F^* hybridomas with one (blue) or two (red) completely rearranged alleles. (**c**) Percent of *Tcrb^A/F^* hybridomas with a single complete VDJb rearrangement on the *Tcrb^A^* (blue) or *Tcrb^F^* (red) allele. (**d**) Flow cytometric analysis of CD4 and CD8 expression in thymocytes from *Tcrb^+/+^*, *Tcrb^A/A^* and *Tcrb^F/F^* mice. Percentages are indicated. Data are representative of at least 4 mice analyzed for each genotype. (**e–f**) Number of DN, DP, CD4SP and CD8SP thymocytes (e) and number of CD4^+^ and CD8^+^ splenic T cells (f) in *Tcrb^+/+^* (blue), *Tcrb^A/A^* (red) and *Tcrb^F/F^* (green) mice, depicted as mean +/− SEM. At least four age-matched mice were analyzed for each genotype.

Efficient assembly and expression of a productive *Tcrb* chain gene is required for the normal development of T cells. Several lines of evidence demonstrate that rearrangement of both the *Tcrb^A^* and *Tcrb^F^* alleles occurs with near normal efficiency. First, in lymph node *Tcrb^A/+^* and *Tcrb^F/+^* T cell hybridomas, complete rearrangements occurred at a high frequency on both the *Tcrb^A^* and *Tcrb^F^* alleles (Fig. 2a and Table S1). Moreover, analysis of *Tcrb^A/A^* and *Tcrb^F/F^* T cell hybridomas revealed that 32% and 35%, respectively, have complete VDJb rearrangements on both alleles (Fig. 2b and Table S2). This is close to the maximum 40% expected due to allelic exclusion [29], [30]. Finally, analysis of *Tcrb^A/F^* T cell hybridomas with single VDJb rearrangements revealed approximately equal frequencies of VDJb rearrangements on the *Tcrb^A^* and *Tcrb^F^* alleles (Fig. 2c and Table S3).

Thymocyte development in *Tcrb^A/A^* and *Tcrb^F/F^* mice was indistinguishable from wild type mice (Fig. 2d and e). In this regard, wild type (*Tcrb^+/+^*), *Tcrb^A/A^* and *Tcrb^F/F^* mice had similar numbers of CD4^−^:CD8^−^ (double negative, DN), CD4^+^:CD8^+^ (double positive, DP) and CD4^−^:CD8^+^ or CD4^+^:CD8^−^ (single positive, SP) thymocytes (Fig. 2d and e). Flow cytometric analysis of Tcrb chain expression revealed no significant differences between *Tcrb^A/A^*, *Tcrb^F/F^* and *Tcrb^+/+^* thymocytes (data not shown). Finally, *Tcrb^A/A^*, *Tcrb^F/F^* and *Tcrb^+/+^* mice have similar numbers of mature CD4^+^ and CD8^+^ splenic T cells (Fig. 2f). Taken together these data demonstrate that the *Tcrb^A^* and *Tcrb^F^* alleles are efficiently rearranged and expressed and can support normal T cell development.

### Differential stability of PTC-containing *Tcrb^A^* and *Tcrb^F^* transcripts

**Figure 3:**
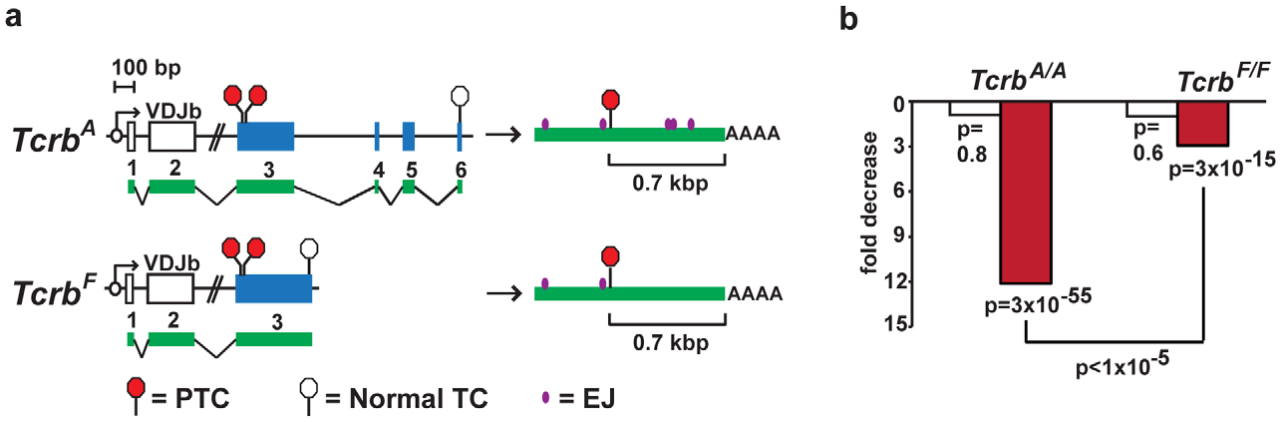
PTC-containing *Tcrb^F^* transcripts show resistance to NMD. (**a**) *Tcrb^A^* and *Tcrb^F^* alleles with completely assembled second exons (VDJb), with four Cb2 exons (blue rectangles 3, 4 5 and 6,*Tcrb^A^*) or the fusion of these exons (blue rectangle 3, *Tcrb^F^*). The positions of the two potential PTCs (red octagons) and the normal termination codon (TC, open octagon) are shown. Pre-mRNAs with exons (green bars) and introns (connecting black lines) are shown, as are completely processed PTC-containing *Tcrb^A^* and *Tcrb^F^* transcripts (green bars) with exon junctions (EJ, purple dots). (**b**) Fold reduction (relative to PTC-containing alleles) in PTC-containing *Tcrb* pre-mRNA (open bars) and completely processed mRNA (red bars) in *Tcrb^A/A^* and *Tcrb^F/F^* DN thymocytes. P-values were calculated by a binomial test for pre-mRNA versus mRNA, and by Monte Carlo simulation for *Tcrb^A/A^* versus *Tcrb^F/F^*.

Like the wild type *Tcrb* locus, *Tcrb^A^* PTCs lie in the third of six exons while *Tcrb^F^* PTCs lie in the third and final exon; thus, *Tcrb^A^* PTCs have downstream introns whereas *Tcrb^F^* PTCs do not (Fig. 3a). Notably, *Tcrb^A^* and *Tcrb^F^* transcript PTCs lie at the same distance (0.7 kbp) from the poly(A) tract and have two upstream introns in the same locations (Fig. 3a). Thus, comparing the stability of PTC-containing *Tcrb^A^* and *Tcrb^F^* transcripts in *Tcrb^A/A^* and *Tcrb^F/F^* thymocytes allows us to determine the relative contribution of mechanisms that rely on introns downstream of the PTC in mediating degradation of PTC-containing *Tcrb* transcripts *in vivo*.

To this end, *Tcrb^A/A^* and *Tcrb^F/F^* CD25^+^ DN thymocytes were purified by flow cytometric cell sorting and VDJb rearrangements utilizing five different Vb gene segments were amplified and sequenced from genomic DNA and cDNAs generated from both *Tcrb* pre-mRNAs and mature transcripts. A total of 1592 sequences were analyzed to identify those that are in-frame (no PTC, PTC^−^) and those that are out-of-frame (PTC-containing, PTC^+^) (Table S4). As compared to genomic DNA, the fraction of PTC^+^ *Tcrb* pre-mRNAs was similar to the fraction of PTC^+^ *Tcrb* alleles in both *Tcrb^A/A^* and *Tcrb^F/F^* DN thymocytes (Fig. 3b, open bars and Table S4). As compared to genomic DNA, there is a twelve-fold reduction in the abundance of PTC^+^ mature *Tcrb^A^* mRNAs (Fig. 3b, red bar and Table S4). In striking contrast, the abundance of PTC^+^ *Tcrb^F^* mRNAs is reduced by only three-fold (Fig. 3b, red bar and Table S4). Thus, PTC-containing *Tcrb^A^* mRNAs are eliminated more efficiently than PTC-containing *Tcrb^F^* mRNAs.

### Concluding Remarks

Here, we show that PTC-containing *Tcrb^A^* and *Tcrb^F^* transcripts have differing abilities to be eliminated in mammalian thymocytes *in vivo*. The only difference between the *Tcrb^A^* and *Tcrb^F^* alleles is the presence of introns downstream of the PTC. Thus, these findings demonstrate that these downstream introns are mechanistically important components in the efficient elimination of PTC-containing *Tcrb* transcripts *in vivo*, consistent with the notion that they are required to activate EJC-dependent NMD. Nevertheless, PTC-containing transcripts templated by the *Tcrb^F^* allele are reduced three-fold in their abundance. Thus, mechanisms that do not rely on downstream introns are also capable of eliminating PTC-containing transcripts, although not to levels achieved when downstream introns are present. As transcripts templated by the *Tcrb^F^* allele will have two introns it is conceivable PTC-containing *Tcrb^F^* transcripts can also be inactivated by nonsense-associated altered splicing or other mechanisms that alter the splicing of PCT-containing transcripts. Notably, PTCs in the endogenous immunoglobulin light chain kappa gene frequently reside in the last exon, and like *Tcrb^F^* transcripts in thymocytes, the abundance of PTC-containing immunoglobulin light chain kappa transcripts is also reduced by about three-fold in developing B cells [19], [31]. We conclude that although PTC-containing *Tcrb* transcripts can be eliminated by several mechanisms in developing lymphocytes *in vivo*, maximal elimination of these transcripts depends on mechanisms that rely on the presence of introns downstream of the PTC.

## Materials and Methods

### Ethics statement

This study was carried out in strict accordance with the recommendations in the Guide for the Care and Use of Laboratory Animals of the National Institutes of Health. The protocol was approved by the Washington University Animal Studies Committee (#20070189).

### Generation of *Tcrb^A/A^ and Tcrb^F/F^* mice

The 5′ homology arm of pLNTK-DJ (Fig. S1) contains a Db2Jb2.3 rearrangement amplified with 5′HDJ and 3′Jb2-3 and the Jb2.4-Jb2.7 gene segments amplified with 5′Jb2.4US and 3′Jb2.7DS (oligonucleotide sequences are listed in Table S5). The Jb2.3 gene segment has a single G to C change to eliminate a PTC generated using the 5′Jb2.3, Jb2.3M1, Jb2.3M2 and 3′Jb2.3X, oligonucleotides. The 3′ homology arm is a 3.2 kbp *ClaI/SpeI* fragment downstream of Jb2.7. The 5′ homology arm of pLNTK-Cb2F (Fig. S2) is a 2 kbp *ClaI/PstI Tcrb* fragment. The 3′ homology arm was generated by amplifying the constant region of a *Tcrb* cDNA with oligonucleotides A through F, 5′Cb2-3′H and 3′Cb2-3′H as shown in figure S2. Deletion of the Db1 gene segment is described elsewhere [32]. Embryonic stem cells (ES) were transfected, selected and injected into C57BL/6 blastocysts as previously described [32]. Intercrossing of *Tcrb^A/+^* and *Tcrb^F/+^* mice led to the expected Mendelian ratios of *Tcrb^A/A^* and *Tcrb^F/F^* mice, respectively.

### Southern blotting

Southern blot analysis of ES cells targeted with pLNTK-DJ was performed on *SacI*-digested genomic DNA with probe A as previously described [33]. For ES cells targeted with pLNTK-Cb2F, *PstI*-digested genomic DNA was probed with a 400 bp *HincII/BamHI* genomic fragment 3′ of the 3′ homology arm, and *SacI*-digested genomic DNA probed with probe A.

### Hybridomas

Hybridomas were generated and *Tcrb* gene rearrangements analyzed as previously described [23].

### Flow cytometric analyses and cell purification

Flow cytometric analyses were performed on a FACSCalibur (BD Biosciences) using FITC-conjugated anti-CD25, PE-Cy5-conjugated anti-CD4 and FITC-conjugated anti-CD8. CD25^+^ DN thymocytes were purified from 4–5 mice for each genotype by flow cytometric cell sorting (FACSVantage BD Biosciences).

### Sequence analyses

Genomic DNA and RNA were isolated as previously described from CD25^+^ DN thymocytes purified by flow cytometric cell sorting [33], [34]. The SuperScriptII Reverse Transcriptase kit (Invitrogen) was used to synthesize cDNA using oligo-dT or the intronic 3′Jb2-11 oligonucleotide for mature mRNA or pre-mRNA, respectively. VDJb rearrangements were amplified from DNA using Vb2, Vb6, Vb8.1, Vb14 or Vb16 and 3′Jb2-6. PCR conditions were 200 ng genomic DNA in 50 uL with 1 mM MgCl_2_, 100 mM dNTPs and 10 picomoles of each oligonucleotide for 17 cycles of 92°C (1∶00), 60°C (1∶30), and 72°C (1∶30), followed by secondary amplification for 30 cycles using 3′Jb2-3 and the same Vb oligonucleotide. cDNA was amplified as above, except using Cb2-1 and Cb2-2 oligonucleotides in the primary and secondary reactions, respectively. The significance of the fold decrease of pre-mRNA or mRNA relative to DNA was calculated using a binomial test. The p-value for fold decrease of mRNA for *Tcrb^A^* versus *Tcrb^F^* was calculated using a Monte Carlo simulation (script available on request).

## Supporting Information

Figure S1 **Targeting strategy for generating the** ***Tcrb^A^*** **allele.** Generation of the *Tcrb^A^* allele. Shown is a schematic of part of the *Tcrb* allele in which the Db1 gene segment has been deleted (top, Jb1^M3^ in ref. [28]). The Db and Jb gene segments are shown as open rectangles (except for Jb2.3, shown as a shaded rectangle) and the RSs as open triangles. The Cb1 and Cb2 exons (blue rectangles) are also shown, as is the pLNTK-DJ targeting vector used to generate the *Tcrb^A+Neo^* allele, which has a targeted replacement of the Db2, Jb2.1, Jb2.2 and Jb2.3 gene segments with a Db2Jb2.3 rearrangement and the *loxP*-flanked neomycin resistance gene (Neo^R^). The *Tcrb^A^* allele generated after Cre-mediated deletion of the neomycin resistance gene, leaving a single *loxP* site (filled oval), is shown. The relative positions of the different restriction sites are shown, as is probe A, which was used for Southern blot analysis of the different targeted alleles. Also shown is a Southern blot of genomic DNA from targeted ES cell lines digested with *SacI* and hybridized to probe A. The molecular weight markers and relative positions of the bands generated by the different *Tcrb* alleles are indicated.
(TIF)

Figure S2 **Targeting strategy for generating the** ***Tcrb^F^*** **allele.** **a**) Generation of the Cb2 fusion. Shown are schematics of the four Cb2 exons (labeled 1 through 4) in genomic DNA and in cDNA generated from completely processed mRNA, and oligonucleotides A through F (labeled arrows). **b**) Generation of the *Tcrb^F^* allele. Shown is a schematic of the Cb2 region of the *Tcrb^A^* allele (top) and the pLNTK-Cb2F targeting vector used to generate the *Tcrb^F+Neo^* allele, which has a targeted replacement of the four Cb2 exons with a DNA fragment containing a fusion of these exons and the *loxP-*flanked neomycin resistance gene. Also shown is the *Tcrb^F^* allele generated after Cre-mediated deletion of the neomycin resistance gene, leaving a single *loxP* site. The relative positions of the different restriction sites are shown, as are probes A and B, which were used for Southern blot analyses. Southern blots of genomic DNA from targeted ES cell lines that were digested with *PstI* and hybridized to probe B, or digested with *SacI* and hybridized to probe A are shown. The molecular weight markers and relative position of the bands generated by the different *Tcrb* alleles are indicated.
(TIF)

Table S1 Number of *Tcrb^A^*, *Tcrb^F^* and *Tcrb^+^* alleles in the VDJb configuration in the *Tcrb^F/+^* and *Tcrb^A/+^* T cell hybridomas analyzed. The total number (n) of hybridomas analyzed is indicated.
(TIF)

Table S2 Number of *Tcrb^A/A^* and *Tcrb^F/F^* T cell hybridomas with *Tcrb* alleles in the VDJb/DJb and VDJb/VDJb configuration.
(TIF)

Table S3 Number of *Tcrb^A/F^* T cell hybridomas with *Tcrb* alleles in the VDJb^A^/DJb^F^ and DJb^A^/VDJb^F^ configuration.
(TIF)

Table S4 Total number of sequences (n) and the number with (PTC^+^) or without (PTC^−^) PTCs from genomic DNA, pre-mRNA and mRNA from *Tcrb^A/A^* and *Tcrb^F/F^* DN thymocytes.
(TIF)

Table S5 Oligonucleotide sequences.
(TIF)
