# Supplementary figures and images for: Out-of-Frame T Cell Receptor Beta Transcripts Are Eliminated by Multiple Pathways In Vivo
Source: PLoS One. 2011 Jul 13;6(7):e21627. doi: 10.1371/journal.pone.0021627 (PMC3135592; doi:10.1371/journal.pone.0021627)

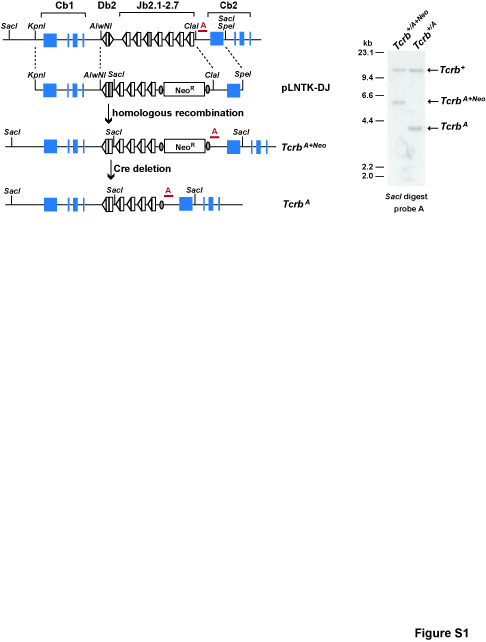

Supplement: Figure S1 — Targeting strategy for generating the TcrbA allele. Generation of the TcrbA allele. Shown is a schematic of part of the Tcrb allele in which the Db1 gene segment has been deleted (top, Jb1M3 in ref. [28]). The Db and Jb gene segments are shown as open rectangles (except for Jb2.3, shown as a shaded rectangle) and the RSs as open triangles. The Cb1 and Cb2 exons (blue rectangles) are also shown, as is the pLNTK-DJ targeting vector used to generate the TcrbA+Neo allele, which has a targeted replacement of the Db2, Jb2.1, Jb2.2 and Jb2.3 gene segments with a Db2Jb2.3 rearrangement and the loxP-flanked neomycin resistance gene (NeoR). The TcrbA allele generated after Cre-mediated deletion of the neomycin resistance gene, leaving a single loxP site (filled oval), is shown. The relative positions of the different restriction sites are shown, as is probe A, which was used for Southern blot analysis of the different targeted alleles. Also shown is a Southern blot of genomic DNA from targeted ES cell lines digested with SacI and hybridized to probe A. The molecular weight markers and relative positions of the bands generated by the different Tcrb alleles are indicated. (TIF) [file pone.0021627.s001.tif]

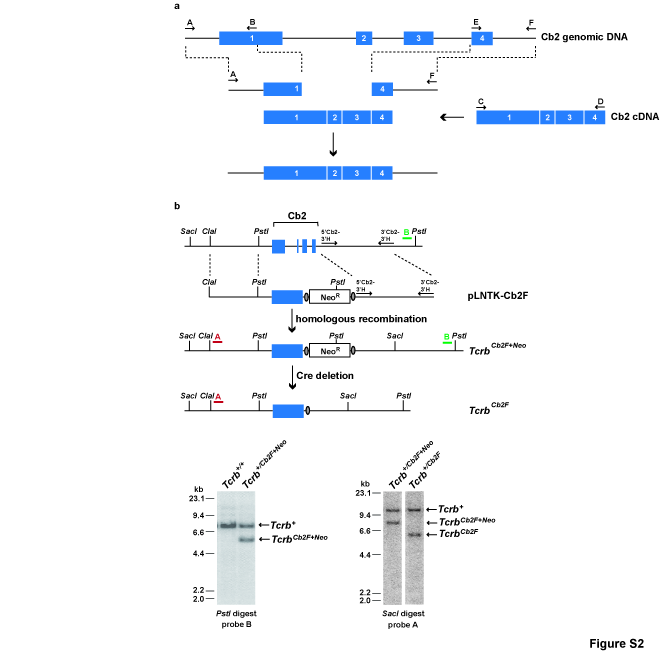

Supplement: Figure S2 — Targeting strategy for generating the TcrbF allele. a) Generation of the Cb2 fusion. Shown are schematics of the four Cb2 exons (labeled 1 through 4) in genomic DNA and in cDNA generated from completely processed mRNA, and oligonucleotides A through F (labeled arrows). b) Generation of the TcrbF allele. Shown is a schematic of the Cb2 region of the TcrbA allele (top) and the pLNTK-Cb2F targeting vector used to generate the TcrbF+Neo allele, which has a targeted replacement of the four Cb2 exons with a DNA fragment containing a fusion of these exons and the loxP-flanked neomycin resistance gene. Also shown is the TcrbF allele generated after Cre-mediated deletion of the neomycin resistance gene, leaving a single loxP site. The relative positions of the different restriction sites are shown, as are probes A and B, which were used for Southern blot analyses. Southern blots of genomic DNA from targeted ES cell lines that were digested with PstI and hybridized to probe B, or digested with SacI and hybridized to probe A are shown. The molecular weight markers and relative position of the bands generated by the different Tcrb alleles are indicated. (TIF) [file pone.0021627.s002.tif]

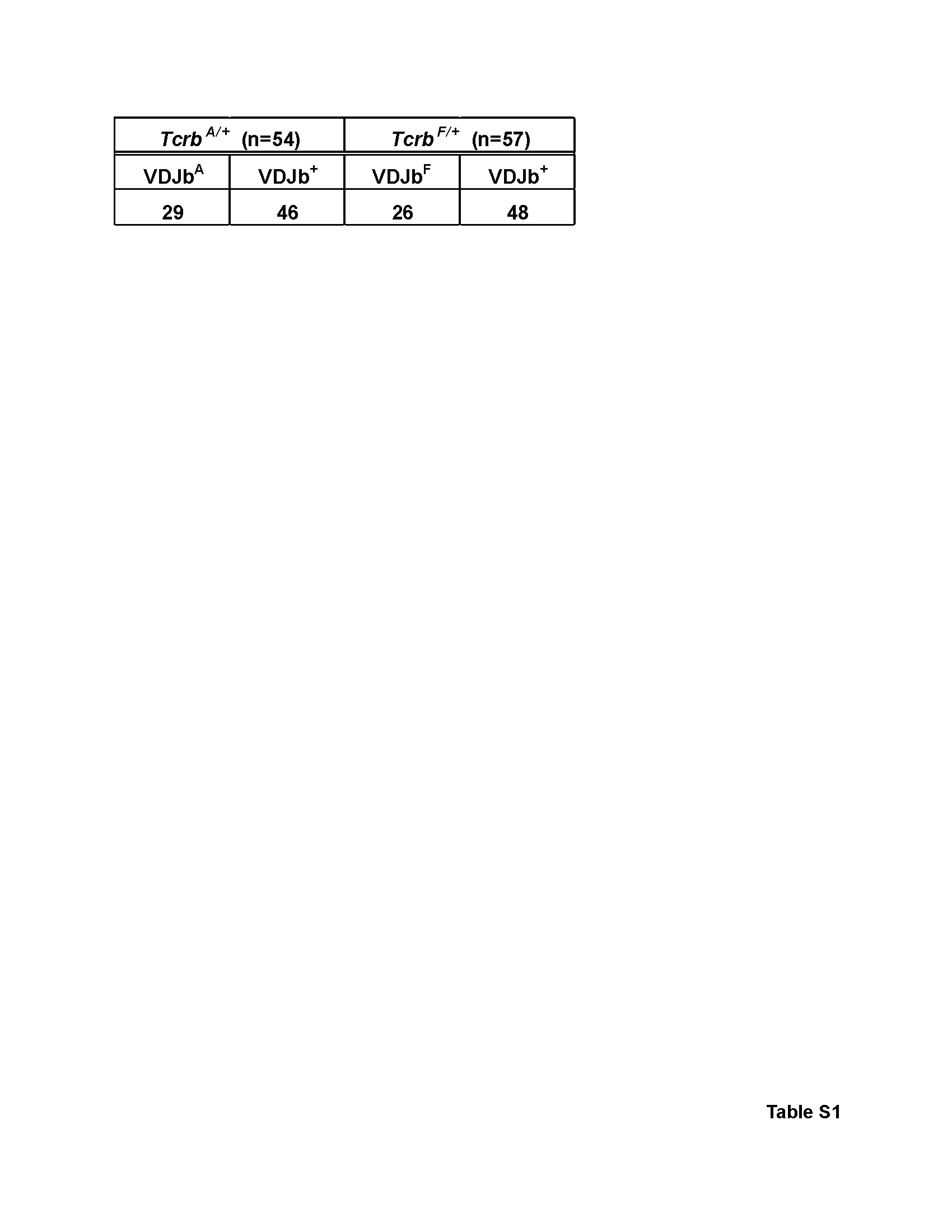

Supplement: Table S1 — Number of TcrbA, TcrbF and Tcrb+ alleles in the VDJb configuration in the TcrbF/+ and TcrbA/+ T cell hybridomas analyzed. The total number (n) of hybridomas analyzed is indicated. (TIF) [file pone.0021627.s003.tif]

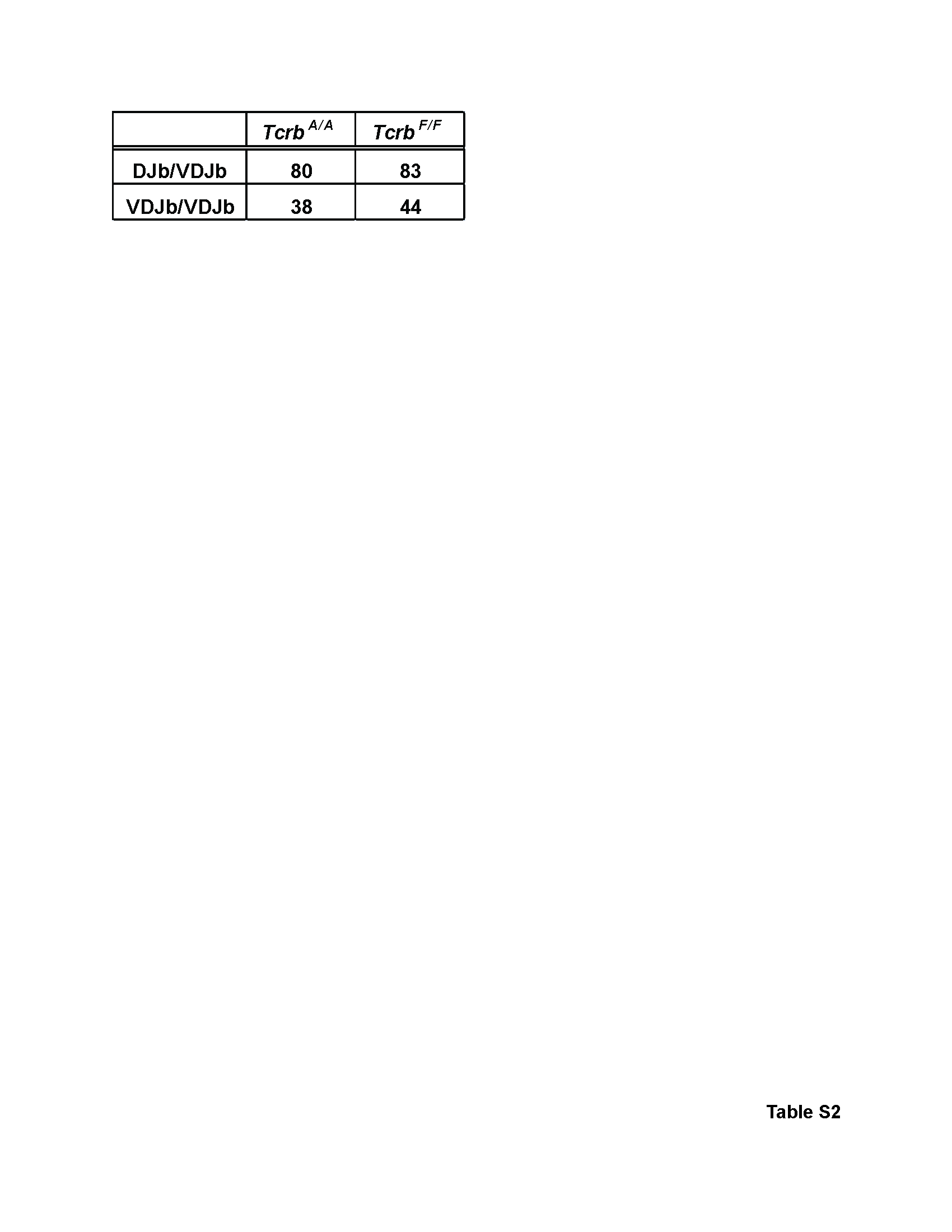

Supplement: Table S2 — Number of TcrbA/A and TcrbF/F T cell hybridomas with Tcrb alleles in the VDJb/DJb and VDJb/VDJb configuration. (TIF) [file pone.0021627.s004.tif]

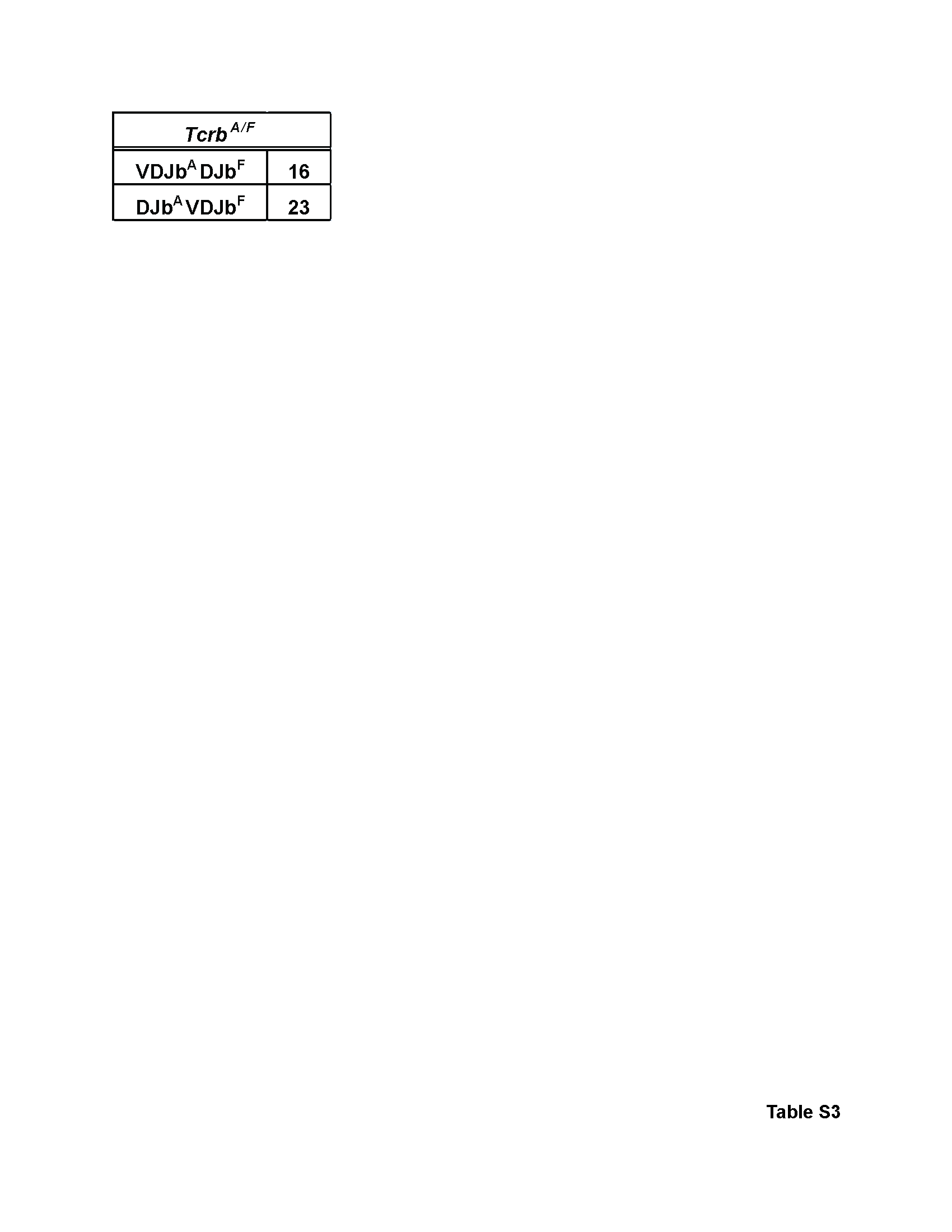

Supplement: Table S3 — Number of TcrbA/F T cell hybridomas with Tcrb alleles in the VDJbA/DJbF and DJbA/VDJbF configuration. (TIF) [file pone.0021627.s005.tif]

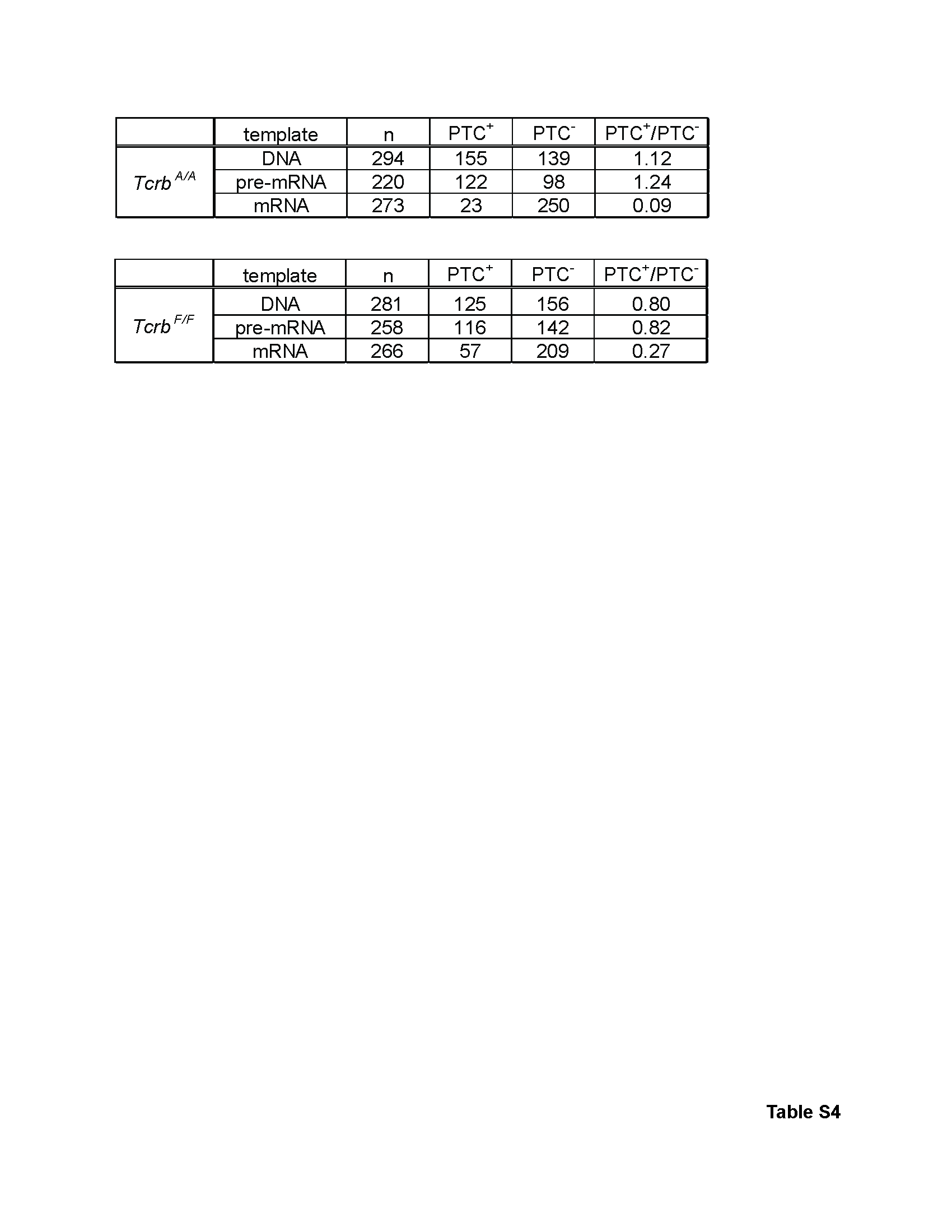

Supplement: Table S4 — Total number of sequences (n) and the number with (PTC+) or without (PTC−) PTCs from genomic DNA, pre-mRNA and mRNA from TcrbA/A and TcrbF/F DN thymocytes. (TIF) [file pone.0021627.s006.tif]

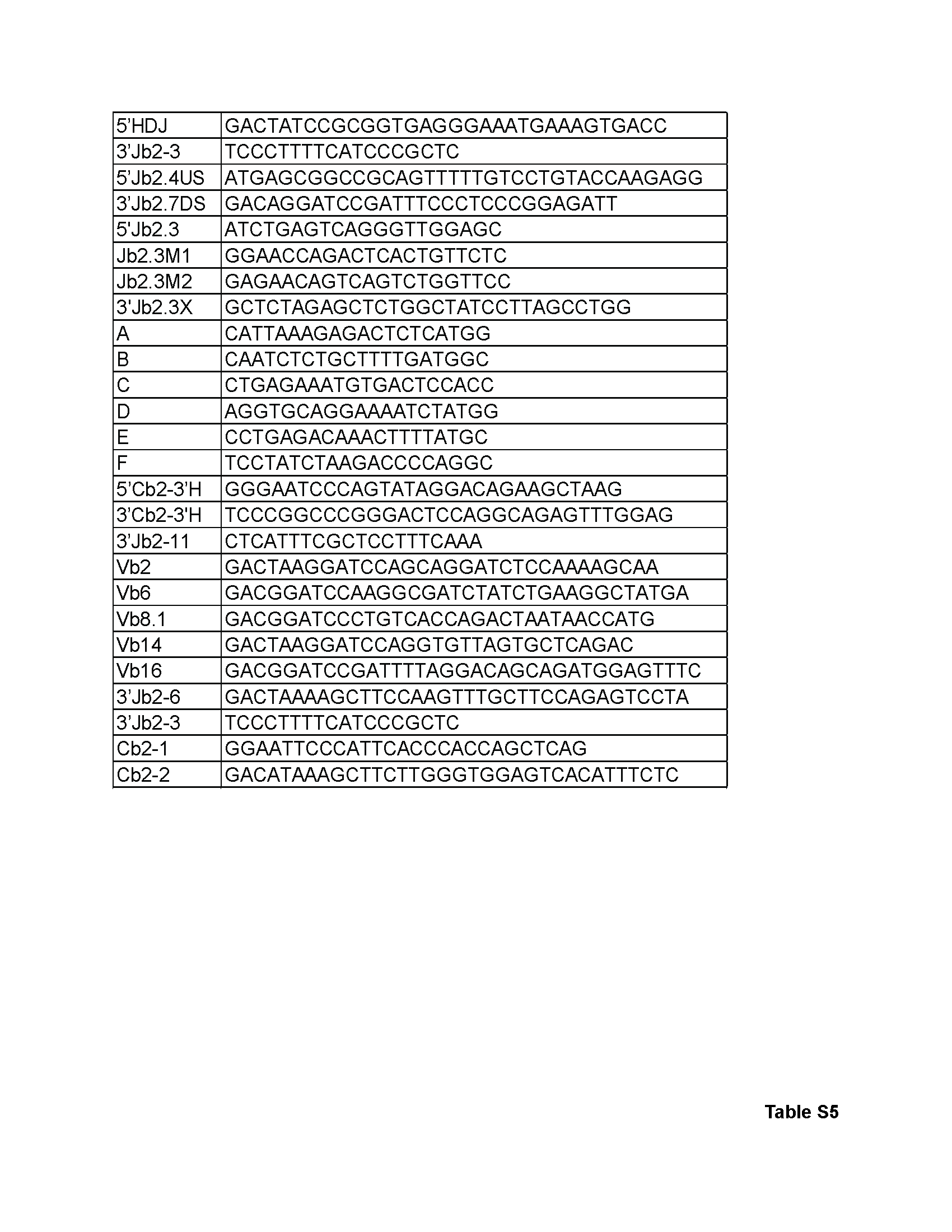

Supplement: Table S5 — Oligonucleotide sequences. (TIF) [file pone.0021627.s007.tif]
